# A robust and tuneable mid-infrared optical switch enabled by bulk Dirac fermions

**DOI:** 10.1038/ncomms14111

**Published:** 2017-01-20

**Authors:** Chunhui Zhu, Fengqiu Wang, Yafei Meng, Xiang Yuan, Faxian Xiu, Hongyu Luo, Yazhou Wang, Jianfeng Li, Xinjie Lv, Liang He, Yongbing Xu, Junfeng Liu, Chao Zhang, Yi Shi, Rong Zhang, Shining Zhu

**Affiliations:** 1 School of Electronic Science and Engineering, Nanjing University, Nanjing 210093, China; 2 Collaborative Innovation Center of Advanced Microstructures, Nanjing University, Nanjing 210093, China; 3 State Key Laboratory of Surface Physics and Department of Physics, Fudan University, Shanghai 200433, China; 4 School of Optoelectronic Information, University of Electronic Science and Technology of China, Chengdu 610054, China; 5 National Laboratory of Solid State Microstructures and School of Physics, Nanjing University, Nanjing 210093, China; 6 Department of Physics, South University of Science and Technology of China, Shenzhen 518055, China; 7 School of Physics and Institute for Superconducting and Electronic Materials, University of Wollongong, New South Wales 2522, Australia

## Abstract

Pulsed lasers operating in the mid-infrared (3–20 μm) are important for a wide range of applications in sensing, spectroscopy, imaging and communications. Despite recent advances with mid-infrared gain platforms, the lack of a capable pulse generation mechanism remains a significant technological challenge. Here we show that bulk Dirac fermions in molecular beam epitaxy grown crystalline Cd_3_As_2_, a three-dimensional topological Dirac semimetal, constitutes an exceptional ultrafast optical switching mechanism for the mid-infrared. Significantly, we show robust and effective tuning of the scattering channels of Dirac fermions via an element doping approach, where photocarrier relaxation times are found flexibly controlled over an order of magnitude (from 8 ps to 800 fs at 4.5 μm). Our findings reveal the strong impact of Cr doping on ultrafast optical properties in Cd_3_As_2_ and open up the long sought parameter space crucial for the development of compact and high-performance mid-infrared ultrafast sources.

## Abstract (web summary)

Mid-infrared pulsed sources are technologically important for sensing and spectroscopy but their implementation is challenging due to the lack of a tuneable optical switch. Here, the authors address this limitation by engineering the band structure of an emerging Dirac semimetal, Cd_3_As_2_.

Short-pulsed lasers have proved indispensable for many branches of science and engineering such as spectroscopy, biomedical research and telecommunications^1^,^2^. The key component to achieve pulsed operation is a passive optical switch, also termed saturable absorber, which can transit between different absorption states on an ultrafast timescale^3^. Although an optical switch may take different physical forms, semiconductor saturable absorber mirrors (SESAMs), a breakthrough in ultrafast photonics in the early 1990s (ref. 4), are at present the most prevalent approach used for ultrashort pulse generation in the near-infrared. Compared with alternative technologies, a most compelling advantage of SESAMs is the ease with which device parameters can be precisely customized with great reproducibility^5^, thanks to the use of mature semiconductor growth techniques, for instance, molecular beam epitaxy (MBE). The design freedom of SESAMs has also opened a desirably large parameter space, enabling the access to robust pulsation regimes and continuous improvement of output characteristics for near-infrared ultrafast lasers^3^.

The development of compact short-pulsed lasers in the mid-infrared has historically been hindered by the poor availability of gain materials, thus expedient techniques based on near-infrared sources and nonlinear frequency conversion have become today's norm for mid-infrared pulse generation^6^,^7^,^8^. The rapid maturing of mid-infrared gain platforms^9^,^10^,^11^ in recent years is calling for saturable absorbers with performance levels on par with their near-infrared counterparts. While being a powerful optical switching solution^12^,^13^,^14^,^15^, SESAMs nevertheless exhibit a relatively narrow nonlinear optical bandwidth and limited long-wavelength access, that is, typically ∼3 μm (ref. 16). Low-dimensional materials, including carbon nanotubes^17^,^18^, graphene^19^,^20^,^21^, transition-metal dichalcogenides^22^, black phosphorus^23^,^24^ and other emerging two-dimensional materials, have been considered for low-cost substitutes for SESAMs^25^,^26^. However, the defect-prone exfoliation and transfer processes inevitably lead to poor repeatability and reliability and it has been challenging to tune the intrinsic nonlinear optical response in these material systems. Therefore, improving the performance levels of current mid-infrared short-pulsed lasers critically depends on the availability of a capable mid-infrared optical switch, preferably with figures of merit on par with those of SESAMs.

Cd_3_As_2_, a representative three-dimensional topological Dirac semimetal (TDS), exhibits stable bulk Dirac states where conduction and valence bands touch at the Dirac nodes and the Dirac fermions disperse linearly along all three momentum directions^27^,^28^. Interband transitions between the two Dirac bands in Cd_3_As_2_ naturally provides a highly robust and amenable light-Dirac fermion-interaction platform, ideally suited for enabling new optical functionalities in the mid-infrared range.

Here, by probing the mid-infrared optical response of bulk Dirac fermions, we show that MBE-grown Cd_3_As_2_ can act as an ultrafast (<10 ps) optical switch in the mid-infrared with operation covering at least the 3–6 μm range. Through an element doping scheme (that is Cr atoms replacing Cd atoms in the compound), flexible tuning of the photocarrier recovery time over an order of magnitude is achieved. Furthermore, the robust parameter customization of Cd_3_As_2_ allows the access to different pulsation regimes in a 3 μm fibre laser in an on-demand manner, pointing to the potential of greatly upgraded performance levels of mid-infrared pulsed lasers. Although various exotic physical phenomena, such as ultrahigh mobility and giant negative magnetoresistance, have been uncovered in three-dimensional TDS systems^29^,^30^,^31^,^32^, our findings show that this emerging class of quantum materials can be harnessed to fill a long known gap in the field of mid-infrared lasers and photonics.

## Results

### Cd_3_As_2_ film growth and characterization

**Figure 1:**
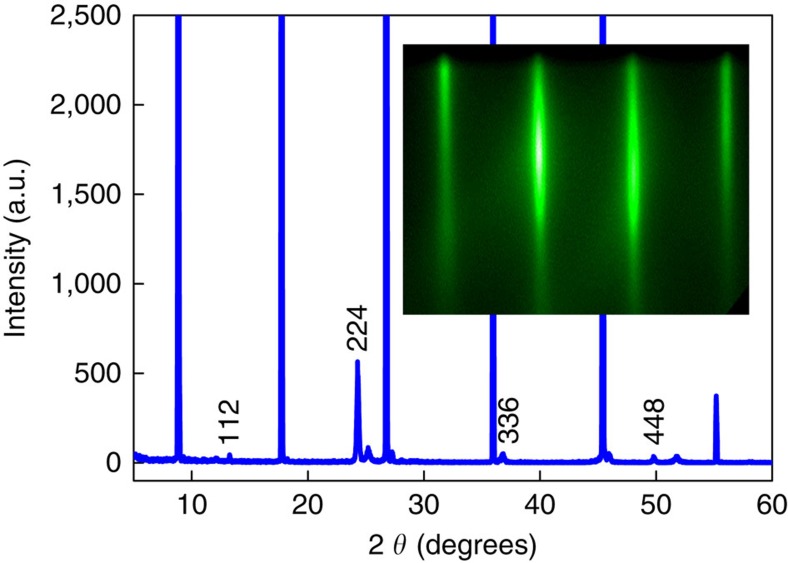
Characterizations of Cd_3_As_2_ thin film. X-ray diffraction pattern for Cd_3_As_2_ thin film sample. The marked peaks correspond to {112} crystal plane of Cd_3_As_2_, while the other peaks come from the mica substrate. The inset is an *in situ* RHEED pattern.

We first prepared high-quality Cd_3_As_2_ films under ultra-high vacuum in MBE system (see Methods section). The thickness of the film was *in situ* monitored by reflection high-energy electron diffraction (RHEED). As shown in Fig. 1 inset, a typical RHEED pattern of Cd_3_As_2_ films shows bright and streaky lines, indicating a good surface morphology and crystallinity. X-ray diffraction measurements were also performed (Fig. 1). A series of peaks can be well resolved and indexed as {112} crystal plane (the un-indexed peaks come from the mica substrate), further confirming the good crystallinity of the sample^33^. Magneto-transport measurements on the Cd_3_As_2_ films were conducted using a physical property measurement system with a superconducting magnet (9 T). The Shubnikov–de Haas oscillations, non-trivial Berry phase and vanishingly small effective mass confirmed the Dirac nature of Cd_3_As_2_ thin film (see Supplementary Fig. 1). Hall measurements revealed an electron mobility of ∼3,300 cm^2^ V^−1^ s^−1^ at room temperature and 5,400 cm^2^ V^−1^ s^−1^ at low temperature (2.5 K).

### Ultrafast nonlinear optical spectroscopy

**Figure 2:**
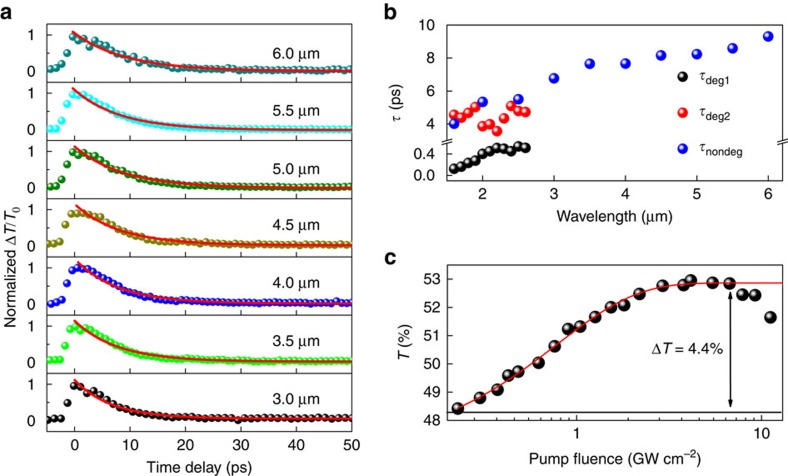
Ultrafast nonlinear optical properties of Cd_3_As_2_ films with 400 nm thickness. (**a**) The non-degenerate ultrafast pump-probe results with the probe wavelength varying from 3 to 6 μm, and red solid lines correspond to a mono-exponential fit for *τ*_nondeg_. (**b**) Fitted relaxation time constants *τ* versus probing wavelengths for both the degenerate (black and red points) and non-degenerate (blue points) measurements. (**c**) The nonlinear absorption at a wavelength of 3 μm (black points) and fitting with a simple saturation model (red line), *T*(*I*)=1−Δ*T* × exp(−*I*/*I*_sat_)−*T*_ns_, where *I*, Δ*T*, *I*_sat_ and *T*_ns_ are the input intensity, modulation depth, saturation intensity and non-saturable absorbance, respectively. A modulation depth of 4.4% and a saturation intensity of ∼0.78 GW cm^−2^ are obtained. The horizontal black line is the base line.

Photoexcitation and carrier relaxation are fundamental processes that govern the optical response of materials. For Cd_3_As_2_, an optical conductivity over broad mid-infrared wavelengths that is linked to the intrinsic Dirac band dispersion provides an important prerequisite for robust and tuneable light–matter interactions^34^. To investigate the ultrafast optical switching characteristics of Cd_3_As_2_, mid-infrared ultrafast pump-probe spectroscopy is performed (see Methods section). It should be noted that, while degenerate probing is known to better approximate saturable absorber dynamics^35^, non-degenerate measurements offer the advantage of convenient scaling of probe wavelengths, owing to the ease of beam alignment (∼6 μm in our setup). Figure 2a and Supplementary Fig. 2a show the non-degenerate and degenerate transient transmission spectra, respectively (a 400 nm thick Cd_3_As_2_ film is used to avoid quantum confinement effects). Both measurements reveal photobleaching signatures arising from Pauli blocking^36^, indicating that Cd_3_As_2_ exhibits saturable absorption over the entire spectral range investigated (1.6–6 μm). We further confirm this nonlinear optical response by a pump fluence-dependent change of Δ*T*/*T*_0_, as shown in Supplementary Fig. 3. A pure mica substrate is found to yield no transient response under the same conditions. Supplementary Fig. 2b illustrates the correlation between the non-degenerate and degenerate photocarrier processes, where *τ*_nondeg_ is seen to reproduce the value of *τ*_deg2_. We therefore attribute the fast component *τ*_deg1_ to the coherent nonlinear optical response resulting from carrier–carrier scattering^37^ and the slow component *τ*_deg2_ (*τ*_nondeg_) to the incoherent response, typically associated with carrier–phonon coupling^38^. In addition, we qualitatively investigate the relative weight of the two relaxation components by plotting the ratio of the slow component's amplitude, defined as Δ*T*/*T*_0_ at 3 ps, to the fast component's amplitude, defined as Δ*T*/*T*_0_ at 0 ps, as a function of probe wavelength. The increasing ratio shown in Supplementary Fig. 2c indicates that the slower, incoherent carrier processes have an increasingly dominant role as the excitation photon energy shifts closer to the Dirac node. Figure 2b summarizes all the fitted time constants for the pump-probe investigation, where *τ*_nondeg_ is seen to slow down from 4 to 9.3 ps as the probe wavelength was increased to ∼6 μm. It should be noted that these time constants are appreciably longer than two-dimensional Dirac fermions in graphene. As theoretical investigations reveal that carrier–acoustic phonon scattering occurs on the microsecond timescale^39^, it is reasonable to attribute the primary relaxation process to optical phonon coupling, and the increase of the recovery time can be accounted for by the reduced optical phonon energy in Cd_3_As_2_ (∼15 meV) with respect to graphene (162–198 meV)^36^,^40^. In brief, these experimental results unambiguously confirm that MBE-grown Cd_3_As_2_ thin films exhibit ultrafast saturable absorption over broad mid-infrared wavelengths from 3 to 6 μm.

To directly reveal the saturable absorption properties of Cd_3_As_2_, we further performed a nonlinear absorption measurement at a wavelength of 3 μm with an optical parametric amplifier (OPA) delivering ∼100 fs pulses. Figure 2c shows the power-dependent transmittance of Cd_3_As_2_. A rollover typically seen in saturable absorbers at high intensity irradiation is observed at fluences exceeding 7 GW cm^−2^ and the sample is found substantially damaged at a fluence of ∼12 GW cm^−2^ (ref. 41). The measurement data can be fitted by a simple saturation model^42^, *T*(*I*)=1−Δ*T* × exp(−*I*/*I*_sat_)−*T*_ns_, where *I*, Δ*T*, *I*_sat_ and *T*_ns_ are the input intensity, modulation depth, saturation intensity and non-saturable absorbance, respectively. This yields a modulation depth of ∼4.4% and a saturation intensity of ∼0.78 GW cm^−2^. At a wavelength of 2 μm, we obtain a modulation depth of ∼3.4%, a saturation intensity of ∼0.25 GW cm^−2^ and a damage threshold of ∼10 GW cm^−2^ with ∼100 fs pulse irradiation (Supplementary Fig. 4a). Similar figures of merit were obtained by using a longer (6 ps) 2 μm pulses from a mode-locked fibre laser (Supplementary Fig. 4b).

### Photocarrier recovery time customization

**Figure 3:**
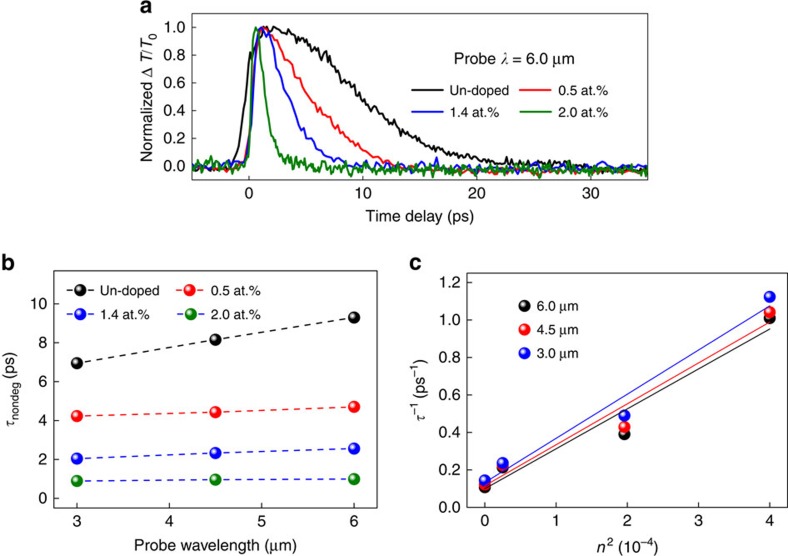
Relaxation time customization by element doping. (**a**) Time-resolved Δ*T*/*T*_0_ traces at a probe wavelength of 6.0 μm for the Cd_3_As_2_ samples with different Cr concentrations, showing faster photocarrier relaxation times for higher Cr concentrations. (**b**) The fitted recovery time constants as a function of Cr concentration at probe wavelengths of 3.0, 4.5 and 6.0 μm. The data points obtained from the same sample are connected by dashed lines to guide the eye. (**c**) Linear relationship between the inverse relaxation time *τ*^−1^ and the square of the doping concentration *n*^2^. The lines (linear fitting) reveal that the scattering rate agrees with the equation 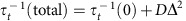, where Δ is the doping-induced band gap and 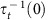 and *D* are constants that are independent of the band gap.

Flexible and precise parameter customization is the most important feature that differentiates SESAMs from other saturable absorber technologies and makes SESAMs adaptable to various laser formats, that is fibre, solid-state or semiconductor chip lasers^3^,^4^,^5^. However, flexible parameter tuning has not been experimentally achieved for mid-infrared saturable absorbers. Among various device parameters, recovery time represents the most fundamental one. Other properties, such as modulation depth and saturation intensity, can typically be controlled by engineering either the recovery time or the device geometry^5^. It should be noted that, for saturable absorber operation, the slow recovery component typically has a more dominant role than the fast component, especially during the initial pulse formation stage^3^. Therefore, various strategies targeting the tuning of the slow component of the relaxation time of SESAMs have been proposed^43^,^44^,^45^, of which low-temperature growth^44^ and postgrowth ion-implantation^45^ have proven most effective. Here we introduce chromium (Cr) as a dopant to the Cd_3_As_2_ film (see Methods section). Compared with low-temperature growth and ion-implantation, one potential advantage of using the Cr doping approach is significantly reduced defects in the sample lattices. In Supplementary Fig. 5, Supplementary Fig. 6 and Supplementary Note 1, we show that the Cr atoms occupy specific (f1) positions instead of distributing randomly (Supplementary Tables 1 and 2), which subsequently leads to the opening of a quasi-particle gap (∼50 meV for the 2% Cr-doped sample)^46^,^47^. The optical conductivity of Cr-doped Cd_3_As_2_ was also calculated (see Supplementary Note 2). As shown in Supplementary Fig. 7, the linear optical absorption is not sensitively dependent on Cr doping for the photon energies of interest. However, the gap opening is expected to lead to strong impact on the photocarrier relaxation dynamics^48^. Figure 3a presents the non-degenerate pump-probe results at 6 μm for Cd_2_As_3_ films with different Cr concentrations (up to 2 at.%). The photocarrier recovery times of Cr-doped Cd_3_As_2_ become appreciably faster at higher Cr concentrations for all wavelengths within 3–6 μm (Supplementary Fig. 8). The time constants as a function of Cr concentration are summarized in Fig. 3b. Without any particular optimization, a relaxation time tuning across an order of magnitude (for example, from 8 ps to 800 fs at ∼4.5 μm) is already achieved, which opens up the long sought-after parameter space for mid-infrared optical switches.

To elaborate the physical mechanisms for the observed relaxation time tuning, we analysed the relaxation dynamics by considering Fermi's golden rule and the Boltzmann equation. It is assumed that the Cr doping opens a gap at the Dirac point, and the gap size is directly proportional to the dopant concentration in the lowest-order coupling^49^. The eigenvector of the current system is shown in Supplementary Note 2. Based on Fermi's golden rule, the carrier relaxation rate (inverse relaxation time) from the initial state |**k**, *s*> to the final state |**k**+**q**, *s*′> is given by^39^,^50^,^51^,^52^,^53^,


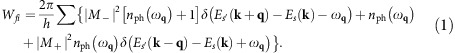


where *n*_ph_ is the phonon distribution function, *ω*_q_ is the phonon energy and *M*_±_ is the electron–phonon interaction matrix element for emitting and absorbing one phonon. The Boltzmann equation for the electron distribution function, 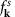, can be written as,


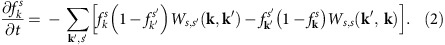


From this equation, the energy loss rate *P* can be calculated^53^, and the relaxation time is given by, *τ*_*t*_=*E*/*P*. The dependence of the relaxation time on the doping concentration can be qualitatively analysed as follows. The interaction matrix element is given as,


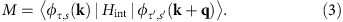


For phonon-mediated carrier relaxation, only two processes contribute to the transition, intranode/interband (11, 11) scattering and internode/interband (11, −11) scattering. The second contribution vanishes in pure Dirac system where the phonon scattering between states from the different nodes are forbidden^54^. However, in the present system, where Cr doping induces a band gap opening, both scattering terms contribute. The scattering matrix elements now include (using the eigenvectors shown in Supplementary Note 2),


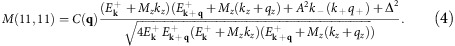



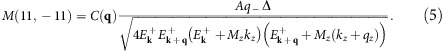


where 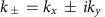 and 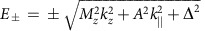. It should be noted that *C*(**q**) depends on the details of electron–phonon interaction, the deformation parameter, electron effective mass and the phonon frequency but is independent of the band gap. Now the scattering rate (inverse scattering time) is the sum of the rate owing to each scattering channels, 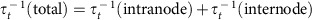. The leading correction in both |*M*(11,11)|^2^ and |*M*(11,−11)|^2^ is proportional to Δ^2^ if higher-order terms are ignored. It is given in the form


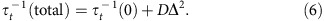


The change of the relaxation rate owing to the gap opening is thus 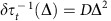. Although both 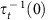 and *D* need to be calculated numerically, the fact that the relaxation rate increases with the band gap size always holds. This band gap dependence has been confirmed in our numerical simulation. To investigate whether our experimental results agree with the above numerical analysis, we plot the experimental inverse relaxation time as a function of the square of the doping concentration *n*^*2*^ in Fig. 3c. Rather good agreement with the theoretical prediction is obtained. From these results, it is reasonable to assign the observed faster relaxation time (at higher Cr concentrations) to additional doping-induced scattering channels as well as the enhancement of intranode scattering, arising from the alteration of the band structure near the Dirac point.

### On-demand pulsed operation in a mid-infrared laser

**Figure 4:**
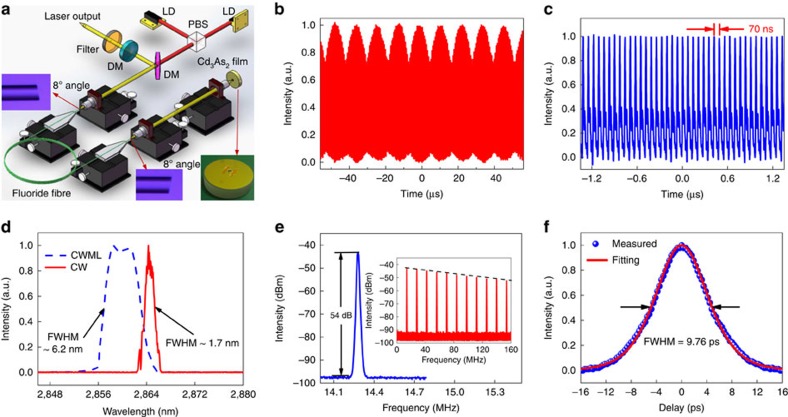
Ultrafast mid-infrared fibre laser based on Cd_3_As_2_ saturable absorber. (**a**) Schematic of the fibre laser setup, where LD, PBS and DM are diode laser, polarized beam splitter and dichroic mirror, respectively. (**b**) *Q*-switched mode-locked pulses at a pump power of 57.6 mW. (**c**) CW mode-locked (CWML) pulses at a pump power of 286.9 mW. There is no discernable envelope modulation, indicating stable operation. (**d**) Output optical spectrum. It is observed that the centre wavelength of CW operation (by moving the focussed beam onto a spot of the gold mirror clear of the Cd_3_As_2_ sample) was red-shifted to 2864.3 nm, as the decreased intracavity loss led to a lower initial Stark manifold of the ^5^*I*_6_ energy level. Meanwhile, the full-width at half-maximum (FWHM) was decreased to 1.7 nm as a result of fewer required spectral Fourier components. (**e**) Radio-frequency (RF) spectrum at a scanning span of 0.8 MHz with a resolution bandwidth of 10 kHz. The repetition rate and signal-to-noise ratio were 14.28 MHz and 54 dB, respectively. The inset shows the RF spectrum with a broader scanning range from 0 to 160 MHz. The smooth roll-off of the clean harmonic frequency components indicated that no *Q*-switched modulation and multiple pulsing were presented in this operation regime. (**f**) Autocorrelation trace measured with an intensity autocorrelator. The blue points are the experimental results and the red line is the fitting result using a Sech^2^ function. The FWHM of the autocorrelation trace is 9.8 ps, corresponding to a pulse duration of 6.3 ps (a deconvolution factor of 1.54 is used to account for the Sech^2^ pulse shape). For **b**–**d**,**f**, the intensities are normalized by the maximum value of measured physical quantities.

To illustrate the benefits of an enlarged parameter space, we demonstrated on-demand access to different pulsation regimes in a home-built 3 μm fluoride fibre laser using Cd_3_As_2_ films with different relaxation times. We chose to employ a fibre laser test bed because it is readily available in our laboratory; the demonstration may be performed on other mid-infrared laser types, such as an extracavity semiconductor laser. Figure 4a shows the pulsed laser setup (see Methods section for details). First, the un-doped Cd_3_As_2_ film with relatively long time constant (∼7 ps at a wavelength of ∼3 μm) was used. When the pump power reached 58 mW, continuous wave (CW) emission turned into *Q*-switched mode-locking envelope as shown in Fig. 4b. With further increasing pump power, both the duration and period of the *Q*-switched envelope decreased as expected for *Q*-switching operation^55^. The *Q*-switched mode-locking state changed swiftly into CW mode-locking at a pump power of 80 mW and could be maintained up to a pump power of 290 mW as shown in Fig. 4c. The pulse period of 70 ns matched well with the calculated cavity round trip time. The optical spectrum of the mode-locked pulses is shown in Fig. 4d. A centre wavelength of 2,860 nm and a full-width half-maximum of 6.2 nm were achieved. Furthermore, the radio-frequency spectra of the pulses were also measured, confirming robust and stable mode-locking operation (Fig. 4e). The pulse duration was measured by a home-built mid-infrared autocorrelator and a pulse width of 6.3 ps was inferred (Fig. 4f). It is clear that stable mode-locking is achieved with the un-doped sample. Then the Cr-doped Cd_3_As_2_ films with shorter relaxation times were introduced into the cavity in turn. It was found that the threshold to achieve CW mode-locking increased with shortening relaxation time of the Cd_2_As_3_, as higher intensities are now required to saturate the conduction band^5^. More specifically, in the case where the Cd_2_As_3_ film with the relaxation time of 0.5 ps (2% Cr doping concentration) was used only the *Q*-switched pulsation regime was accessible as shown in Supplementary Fig. 9. The simple adjustment of the mid-infrared optical switch is expected to greatly facilitate more thorough studies of pulsation regimes of various mid-infrared lasers. It should be noted that, although the technical characteristics of the demonstrated mode-locked laser are similar to those enabled by SESAMs or low-dimensional materials (such as graphene and black phosphorus)^56^,^57^,^58^,^59^,^60^, Cd_3_As_2_ possesses advantages in terms of scaling to longer mid-infrared wavelength as well as flexibility in customizing the relaxation time.

## Discussion

It is worth pointing out that the high level of parameter customization of Cd_3_As_2_ has broad implications. For example, quantum cascade lasers (QCLs) provide an excellent gain platform in the mid-infrared range^11^,^61^,^62^,^63^. However, up until now, robust mode locking of QCLs is only achievable through active mode locking techniques^64^,^65^,^66^,^67^. Our findings make it possible to verify whether mid-infrared QCLs might be passively mode-locked, a subject that can only be fully investigated with a flexibly configurable optical switch.

In conclusion, we have demonstrated a highly robust and tuneable mid-infrared optical switch based on the emerging three-dimensional Dirac semimetal Cd_3_As_2_. Owing to the strong light–matter interaction of bulk Dirac fermions and the compatibility with MBE growth, the use of Cd_3_As_2_ ensures synthesis scalability, broadband operation and flexible parameter control. These features effectively make the Cd_3_As_2_-based approach a capable mid-infrared counterpart to the highly adaptable near-infrared SESAMs. Our work represents a step forward in the development of compact mid-infrared ultrafast sources for advanced sensing, communication, spectroscopy and medical diagnostics. It may be further extended to active photonic devices, including optical modulators and light-emitting devices, working in the mid- to far-infrared range.

## Methods

### Cd_3_As_2_ film growth

A series of Cd_3_As_2_ thin films were grown in a CREATEC MBE system with base pressure <2 × 10^10^ mbar. The substrates were degassed at 350 °C for 30 min to remove any molecule that was absorbed on the mica substrates prior to the growth. The Cd_3_As_2_ thin film deposition was carried out by co-evaporating high-purity Cd (99.999%) and As (99.999%) from dual-filament and valve-cracker effusion cells, respectively. The beam flux ratio of Cd to As was fixed around 3, and the growth process was *in situ* monitored by RHEED. For Cr doping, another cell with Cr (99.999%) was used for co-evaporation and the doping concentration was precisely controlled by adjusting the cell temperature, and the doping concentration is calibrated by energy-dispersion X-ray spectroscopy inside a scanning electron microscope.

### Pump-probe measurement

Both the degenerate and the non-degenerate pump-probe setup is based on an 800 nm, 1 kHz Ti: Sapphire amplifier system (Libra, Coherent Inc.). For the non-degenerate pump-probe experiment, a portion of the laser output energy with a wavelength of 800 nm is used to excite photocarriers in the sample, and the remaining part is fed into an optical parametric amplifier (OPA-SOLO, Coherent Inc.) to generate probe beam with wavelengths from 1.6 to 6 μm. For the degenerate pump-probe measurement, the idler beam (1.6–2.6 μm) of the OPA is split into pump and probe. Both pump and probe pulses have durations of ∼100 fs. For all the measurements, the used pump fluence is about 300 μJ cm^−2^, except for the pump fluence-dependent measurement. In addition, the pump fluence is 20 times larger than the probe fluence. The pump-induced change of probe is detected using a lithium tantalate pyroelectric detector (DET-L-PYC5-R-P, Newport) photodetector and a lock-in amplifier referenced to a 500 Hz chopped pump.

### Nonlinear absorption measurement

The 2 and 3 μm femtosecond pulses (∼100 fs) were generated by the same OPA system used in the pump-probe measurements, and picosecond pulses (∼6 ps) were obtained from a mode-locked thulium fiber laser (NPI Lasers, Inc.) operating at 1,950 nm with 32 MHz repetition.

### 3 μm fibre laser setup

The pulsed fibre laser setup is described in Fig. 4a. Two commercially available diode lasers (Eagleyard Photonics, Berlin) centred at ∼1,150 nm were employed to pump the gain fibre after polarization multiplexing via a polarized beam splitter (PBS) and then focussed by an antireflection-coated (for ∼1150, nm) ZnSe objective lens (Innovation Photonics, LFO-5-6-3 μm, 0.25 NA) with a 6.0 mm focal length. Note that this objective lens also acts as the collimator of the light out-coupled from the fibre core. A dichroic mirror with ∼96% transmittance at 1150, nm and 95% reflectance at ∼3 μm was placed between the PBS and ZnSe objective lens at an angle of 45° with respect to the pump beam to direct the laser. Another specifically designed dichroic mirror with 80% reflection at ∼3 μm was used to act as output coupler. A 3 μm filter with a full-width half-maximum of 500 nm was used to block the residual pump. The gain fibre (Fiberlabs, Japan) was a piece of commercial double-cladding Ho^3+^/Pr^3+^ co-doped fluoride fibre. It has an octangular pump core with a diameter of 125 μm and NA of 0.5 and a circular core with a diameter of 10 μm and NA of 0.2. The concentration of the Ho^3+^ and Pr^3+^ were 30,000 and 2,500 p.p.m., respectively, thus the selected fibre length of 6.8 m provided >90% pump absorption efficiency. Both ends of the fibre were cleaved at an angle of 8° to avoid parasitic lasing in the cavity. First, the laser from the angle-cleaved fibre end far from the pump source was collimated employing a ZnSe objective lens with a specifically designed coating (Innovation Photonics, LFO-5-12-3 μm, 0.13 NA) with a focal length of 12 mm (>95% transmission at 3 μm, and <10% transmission at 1150, nm). Then the collimated light was focussed with a second identical ZnSe objective lens onto the terminated feedback assembled by pasting the Cd_2_As_3_ films on a commercial gold-protected mirror (Thorlabs), as shown in the inset of Fig. 4a. Here the terminator was mounted onto a high-precision six-dimension adjuster to perform position optimization.

### Data availability

All important data supporting the findings of this study are included in this published article (and its Supplementary Information files). Further data sets are available from the corresponding author on reasonable request.

## Additional information

**How to cite this article:** Zhu, C. *et al*. A robust and tuneable mid-infrared optical switch enabled by bulk Dirac fermions. *Nat. Commun.* **8**, 14111 doi: 10.1038/ncomms14111 (2017).

**Publisher's note:** Springer Nature remains neutral with regard to jurisdictional claims in published maps and institutional affiliations.

## Supplementary Material

Supplementary Information Supplementary Figures, Supplementary Tables, Supplementary Notes and Supplementary References
