# Supplementary material for: A robust and tuneable mid-infrared optical switch enabled by bulk Dirac fermions
Source: Nat Commun. 2017 Jan 20;8:14111. doi: 10.1038/ncomms14111 (PMC5263875; doi:10.1038/ncomms14111)
Supplement: Supplementary Information — Supplementary Figures, Supplementary Tables, Supplementary Notes and Supplementary References [file ncomms14111-s1.pdf]

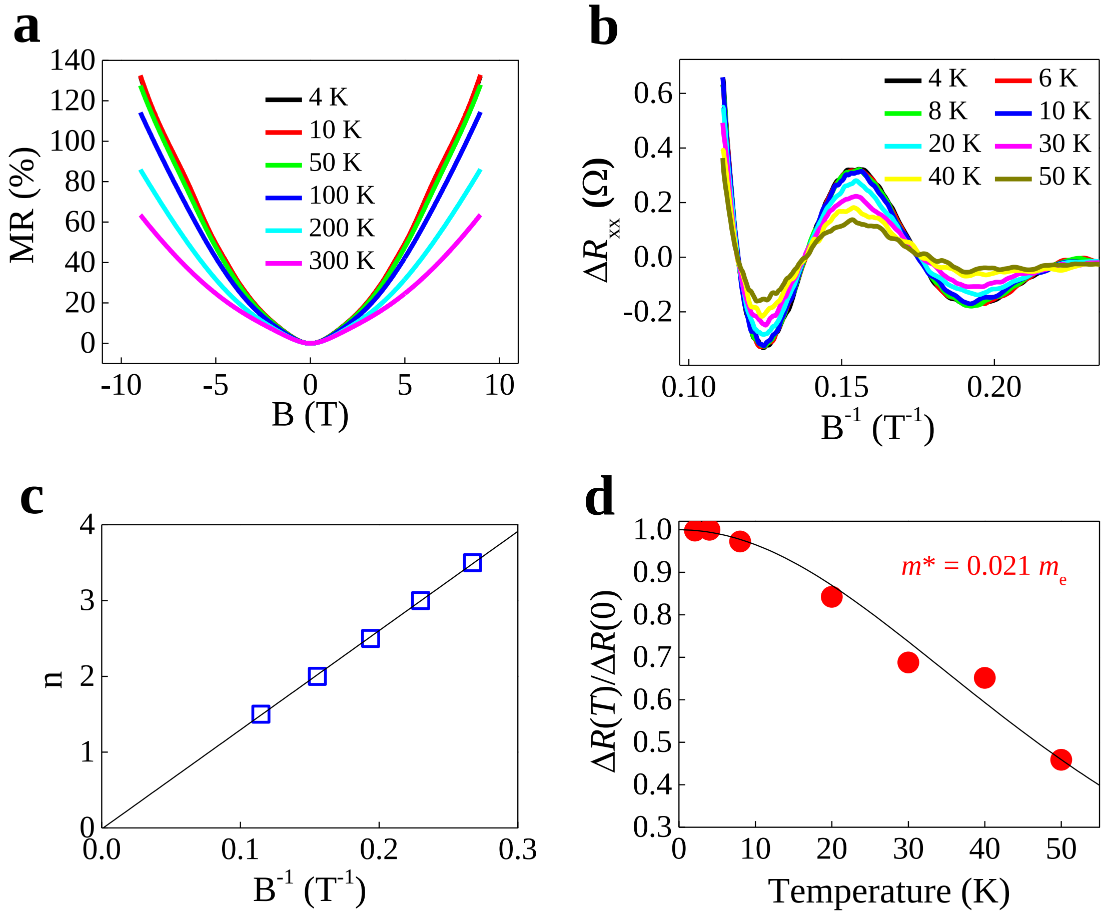

Supplementary Figure 1 | Magneto-transport of Cd_3As_2 thin films. (**a**) Magnetoresistance (MR) at different temperatures under perpendicular magnetic field. At low temperature, the MR ratio reaches 130 % within 9 T. (**b**) Extracted temperature-dependent Shubnikov-de Haas (SdH) oscillations (without MR background). (**c**) A Landau fan diagram. By performing a linear fit (solid line), the intercept is derived as -0.04±0.06, corresponding to the non-trivial Berry phase. This is a distinct feature of ultra-relativistic Dirac fermions which is shared by topological insulators[1], graphene[2] and single crystal bulk Cd_3As_2[3]. (**d**) Temperature-dependent oscillation amplitude. The cyclotron mass of the system can be estimated based on the formula $\Delta R(T) = \Delta R(0)/\sinh(\lambda(T))$, where the thermal factor $\lambda(T)$ is given by $\lambda(T) = 2\pi^2 k_B T m^*/\hbar e B$ and k_B and m^* are Boltzmann's constant and cyclotron mass, respectively. Best fitting to the data in panel **d** gives an effective mass of m^*=0.021m_e, where m_e is the free electron mass. The vanishingly small effective

mass further confirms the Dirac nature of Cd_3As_2 thin films.

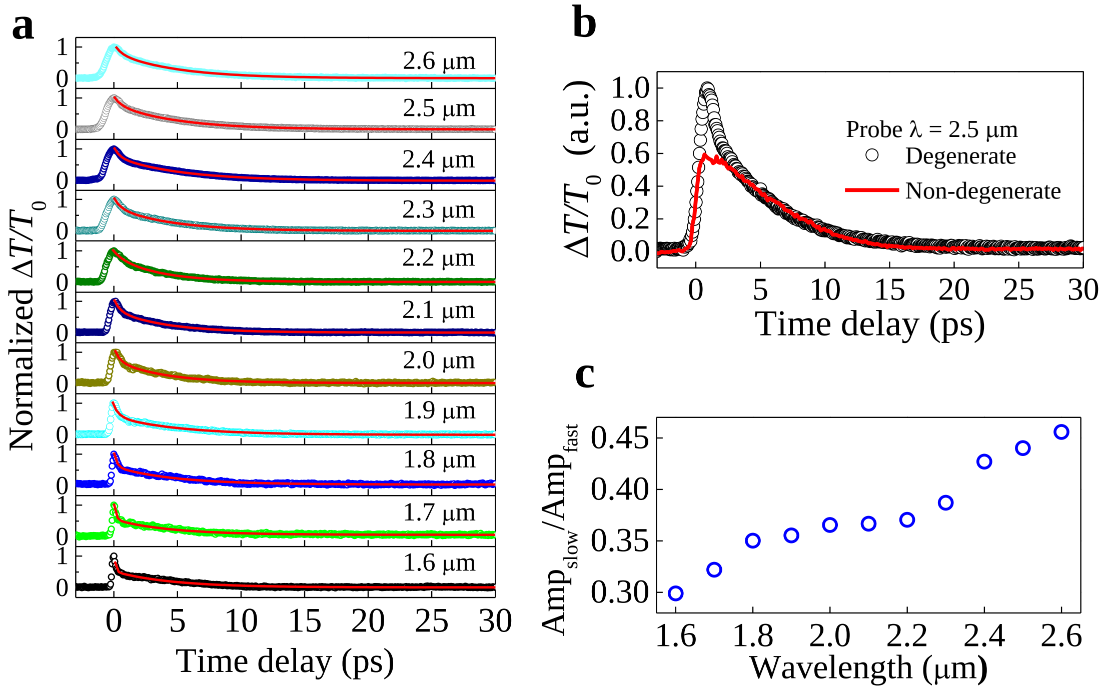

Supplementary Figure 2 | Degenerate ultrafast spectroscopy of Cd_3As_2 films. (**a**) The degenerate results for Cd_3As_2 film with the wavelength range from 1.6 to 2.6 μm, showing two relaxation components. The red solid lines correspond to a bi-exponential fit. (**b**) Time-resolved differential transmission spectra at 2.5 μm, showing the correlation between degenerate and non-degenerate relaxation processes. (**c**) The ratio of the slow component's amplitude ($\Delta T/T_0$ at 3 ps) to the fast component's amplitude ($\Delta T/T_0$ at 0 ps). The increasing ratio with the increase of probe wavelength suggests that incoherent optical response, as governed by phonon effects, plays a more important role in the dynamical photocarrier processes when the excitation photon energy is close to the Dirac node.

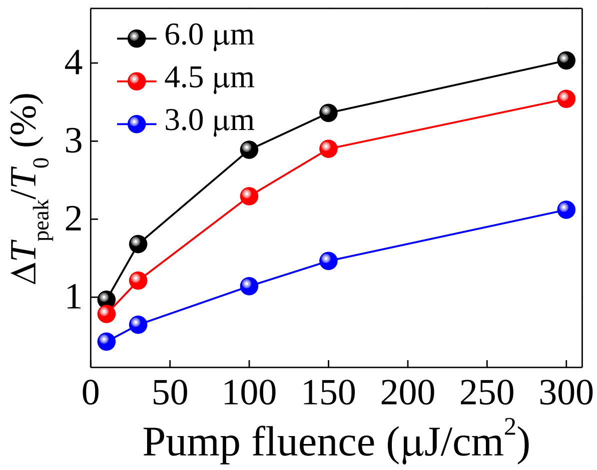

Supplementary Figure 3 | Pump fluence dependent measurements. The peak value of $\Delta T/T_0$ as a function of pump fluences for an un-doped Cd_3As_2 film sample with 800 nm pump and 3 μm, 4.5 μm and 6 μm probe.

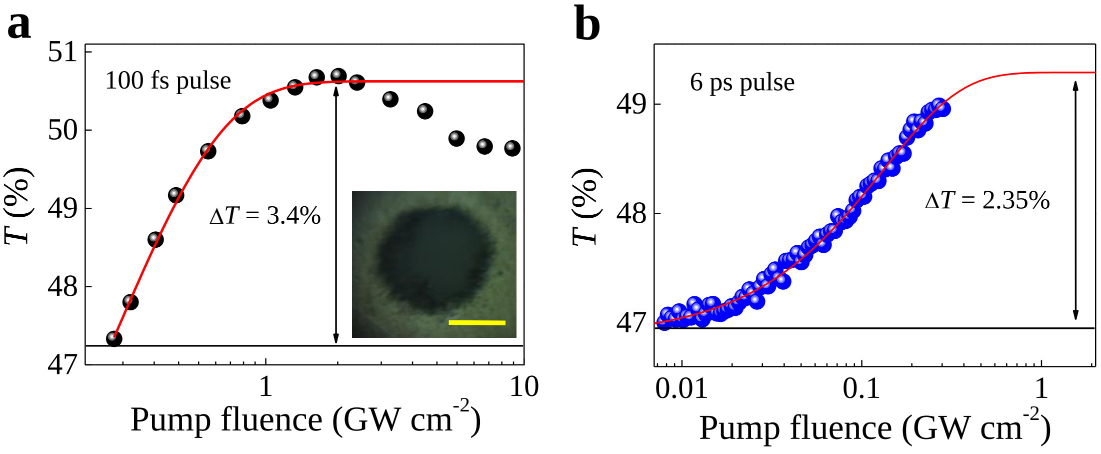

Supplementary Figure 4 | The nonlinear absorption curves at a wavelength of 2 μm for an un-doped Cd_3As_2 film with 400 nm thickness. (**a**) A modulation depth of 3.4% and saturation intensity of ~0.25 GW cm^{-2} can be obtained with ~100 fs pulse irradiation. The damage threshold of a Cd_3As_2 film at 2 μm is about 10 GW cm^{-2}. The inset shows damage morphology of Cd_3As_2 film, and the scale bar is 100 μm. (**b**) A modulation depth of ~2.35% and saturation intensity of ~120 MW cm^{-2} can be obtained with ~6 ps pulse irradiation.

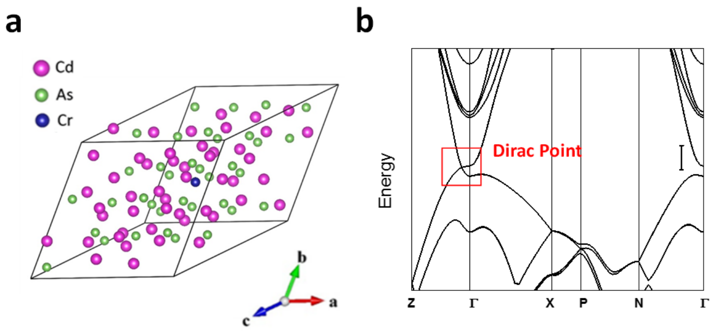

Supplementary Figure 5 | Band structure calculations based on density-functional theory. (**a**) The crystal structure and atom position in x ~ 2% doped Cd_3As_2. (**b**, **c**) Calculated band structures of the un-doped and ~ 2% Cr-doped Cd_3As_2, respectively. The scale bar is 100 meV.

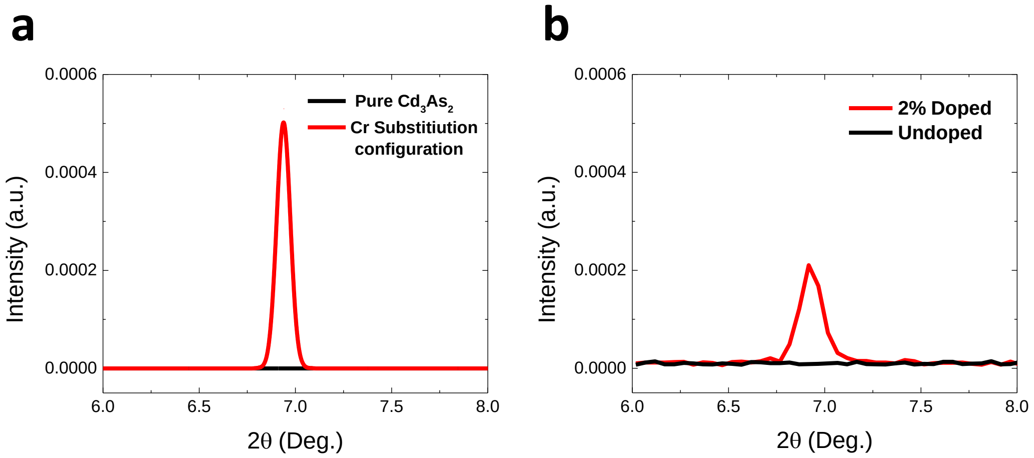

Supplementary Figure 6 | Fine structure characterizations by X-ray diffraction. (**a**) Calculations based on density-functional theory for the given substitution configuration (one Cr atom at f1 position). (**b**) Experimental X-ray diffraction spectra. The intensity is normalized by the intensity of (224) peak.

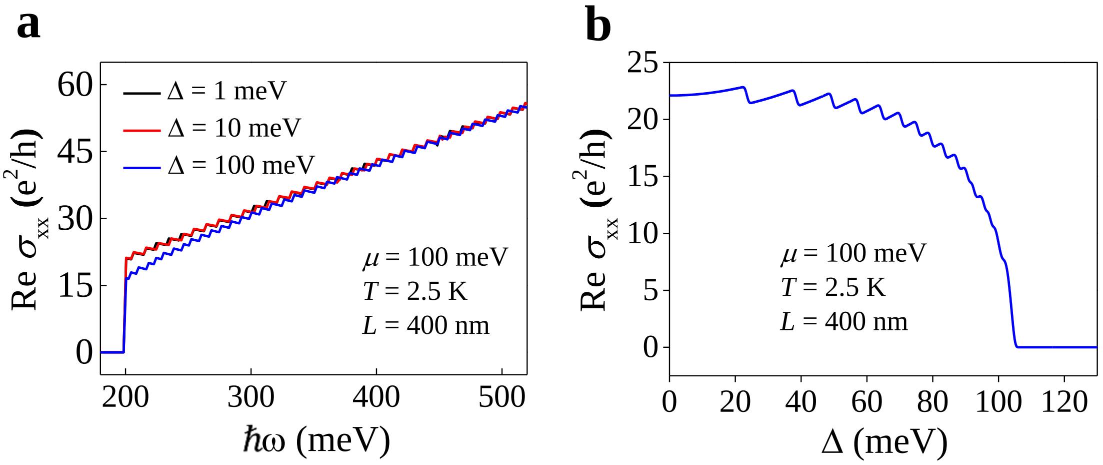

Supplementary Figure 7 | The linear optical absorption of Cd_3As_2 thin films. (**a**) Frequency dependent linear absorption of Cd_3As_2 thin film for three different gaps. (**b**) The gap dependent linear absorption of Cd_3As_2 thin film at a photon energy of 210 meV. Fermi energy μ=100 meV agrees with Fourier transform infrared spectrum (FTIR) measurement, and temperature (T) and film thickness (L) were set to 2.5 K and 400 nm, respectively. The weak oscillation in the curves are due to the thin film model used in our simulation. In the thin film model, the wavevector k_z is quantised. As a result the linear absorption jumps when the phonon energy moves across an additional energy level in the transverse direction. Such weak oscillation will be smeared out by high temperature and by disorder.

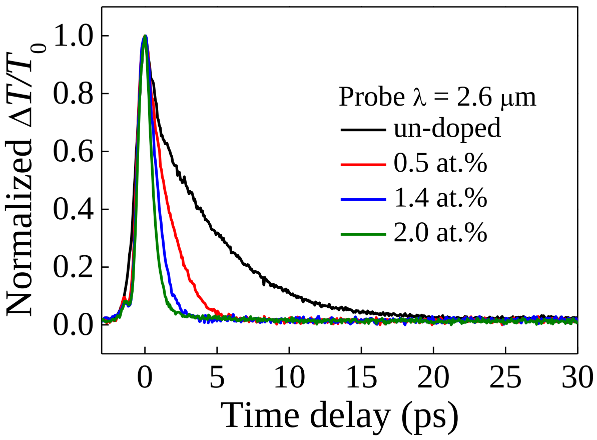

Supplementary Figure 8 | Relaxation time characterizations by the degenerate measurements. Degenerate transient absorption ($\Delta T/T_0$) curves at a probe wavelength of 2.6 μm for the Cd_3As_2 samples with different Cr concentrations. Similar to the non-degenerate results in the main text, the decay rates of photoexcited carriers become faster at high Cr concentrations.

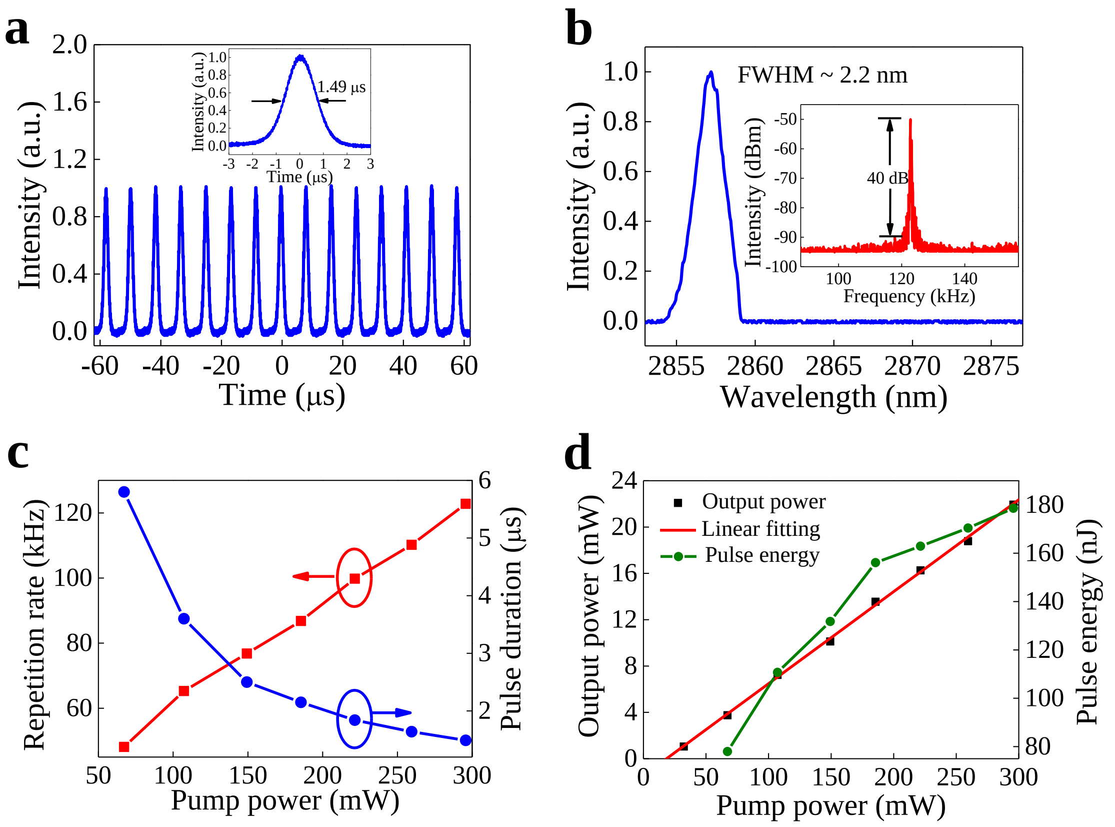

Supplementary Figure 9 | Passively *Q*-switched operation using Cd_3As_2 film with 2% Cr doping concentration. (**a**) *Q*-switched pulse train at a pump power of 295.7 mW. Low Amplitude fluctuation and inconspicuous temporal jitter indicated stable *Q*-switched operation. Inset is the single waveform with gives full width at half maximum (FWHM) of 1.49 μs. (**b**) Output optical spectrum with the centre wavelength of 2857.2 nm and FWHM of 2.2 nm at the pump power of 295.7 mW. Inset is the radio-frequency (RF) spectrum at a scanning span of 70 kHz with a resolution bandwidth of 100 Hz. The signal-to-noise of 40 dB is also located at the typical range of stable *Q*-switching. (**c**) Pulse duration and repetition rate as a function of the pump power. It is observed that the repetition rate increases almost linearly from 48.1 kHz to 122.8 kHz with the launched pump increasing from 67.2 mW to 295.7 mW. Meanwhile, the pulse duration decreases nonlinearly from 5.8 μs to 1.49 μs. Both of them are the typical features of passively *Q*-switched lasers. (**d**) Output power and pulse energy as a function of pump power. The threshold of

Q-switching is 67.2 mW. The pulsed output power increases almost linearly from 3.75 mW to 21.93 mW with a fitted slope efficiency of 7.9% at the pump range from 67.2 mW to 295.7 mW. Meanwhile the pulse energy increases nonlinearly from 77.9 nJ to 178.6 nJ.

Supplementary Table 1 Atom ratio of Cr-doped Cd_3As_2 samples with different Cr concentrations. The doping concentration is obtained by energy-dispersion X-ray spectroscopy inside a scanning electron microscope.

	Cr	Cd	As
**1**	0.0	60.3	39.7
**2**	2.1	58.4	39.5
**3**	4.6	55.1	40.3
**4**	5.9	54.5	39.6

Supplementary Table 2 The calculated system energy with different substitution position

Position	a1	b1	c1	d1	e1	f1
**Coordinate *x***	0.501	0.255	0.549	0.369	0.652	0.422
**Coordinate *y***	0.524	0.278	0.263	0.655	0.627	0.400
**Coordinate *z***	0.735	0.260	0.500	0.503	0.734	0.262
**Energy (meV)**	168	63	246	248	171	0

Supplementary Note 1. DFT Calculations

We performed density-functional theory (DFT) calculations on both un-doped and Cr-doped Cd_3As_2. Experimentally, we found that Cd atoms are substituted by Cr dopants, through energy-dispersion X-ray spectroscopy measurements (Supplementary Table 1). In pure Cd_3As_2, there are 6 distinct nonequivalent position types for Cd, and each type contains 8 different positions in a primitive unit cell. First, we calculate the electronic structure of the pure Cd_3As_2, which agrees well with previous reports where a Dirac cone along Γ line is clearly seen (Supplementary Fig. 5b). Then we substitute one Cr for one Cd atom in a primitive unit cell, which approximates the case for ~2% concentration (Supplementary Fig. 5a). Configurations with the Cr atom in the same position type is equivalent, so it has only 6 nonequivalent configurations here. We calculate the total energy of doped Cd_3As_2 with the Cr atom in non-equivalent position a1-f1 (see Supplementary Table 2), respectively. The most stable configuration corresponds to the scenario where a Cr atom occupies the f1 position, and the calculated energy difference among these possible configurations is quite large (Supplementary Table 2). From the table, one expects that the Cr atoms should occupy specific (low energy) positions instead of distributing randomly. Thus, the substitution of Cd atoms will automatically break the C_4 rotational symmetry around k_z axis. Without the rotational symmetry protection, the Dirac nature and band topology are significantly altered. The calculated C_4 rotation symmetry breaking and the site substitution can be experimentally verified by examining the fine structure though X-ray diffraction (XRD) spectroscopy.

Based on the most likely geometry (substitution at f1 position), a doping induced peak around seven degrees is predicted. The peak intensity is also predicted to be 2000 times smaller

than the main (224) peak. This prediction is realized by the XRD experiments. As shown in Supplementary Fig. 6, the measured peak shows almost identical position but the intensity is 2 times smaller although still on the same order of magnitude. The lower intensity indicates that the doping induced substitution is neither ideal nor perfectly repeated in each unit cell. However, the observed fine structure at the right diffraction angle can clearly verify the site substitution and the resultant C_4 rotation symmetry breaking. If the Cr atoms are randomly distributed, no peak should appear on the XRD spectrum in the doped sample.

Supplementary Fig. 5c displays the electronic structure of Cr-doped Cd_3As_2 with one of the Cd atoms in unit cell substituted by a Cr atom. Due to the broken rotation symmetry, the original Dirac cone is eliminated by the generation of a finite quasi-particle gap which directly supports the existence of massive fermions. Therefore, combining the DFT calculations and high-resolution XRD results, it can be concluded that the Cr doping leads to a symmetry breaking and band structure modification.

Supplementary Note 2. Optical conductivity in Cr-doped Cd_3As_2

Models and eigenstates. The general form of the low-energy Hamiltonian for a Weyl semimetal is described as[4,5],

$$H = H_0 + H_{\mathrm{int}}. \quad (1)$$

where

$$H_0 = \int d^3r \left[v_{\mathrm{f}} \psi^\dagger(\mathbf{r}) \begin{pmatrix} \sigma \cdot (-i\nabla - \mathbf{b}) & 0 \\ 0 & -\sigma \cdot (-i\nabla - \mathbf{b}) \end{pmatrix} \psi(\mathbf{r}) - \mu_0 \psi^\dagger(\mathbf{r}) \psi(\mathbf{r}) \right]. \quad (2)$$

Here σ denotes the Pauli spin matrices, ψ is the field operators, μ_0 is the mass term which can be regarded as arising from the internode coupling, or the bare gap. From this general Hamiltonian,

we made two modifications: **b**=0 for Dirac semimetals, and the dispersion along the z direction is different to that along the film surface. Furthermore, the z-component of the electron momentum is quantized. The bare gap term can be written as[4] $\gamma^0\mu_0$, where the γ^0 matrix is given as

$$\gamma^0 = \begin{pmatrix} 0 & -I \\ -I & 0 \end{pmatrix}. \quad (3)$$

The interaction term H_{int} is the electron-phonon coupling which governs the relaxation of photo-excited hot carriers.

For linear interband absorption (vertical transition), we only consider H_0, which is now given as

$$H_0 = \begin{pmatrix} M_z k_z & Ak_- & \Delta & 0 \\ Ak_+ & -M_z k_z & 0 & \Delta \\ \Delta & 0 & -M_z k_z & -Ak_- \\ 0 & \Delta & -Ak_+ & M_z k_z \end{pmatrix}. \quad (4)$$

where $k_\pm = k_x \pm ik_y$, $M_z = 2M_1 k_D$, with $k_D = \sqrt{M_0/M_1}$. The spectrum is described as $E_\pm = \pm\sqrt{M_z^2 k_z^2 + A^2 k_{||}^2 + \Delta^2}$. From the experimental result of $m* = 0.021m_e$, the estimated bare gap is 0.6 meV. For low doping concentration in the Born approximation, the gap is proportional to the doping concentration.

The eigenstates are labelled by two quantum number $\tau = \pm 1$, $\sigma = \pm 1$.

$$|\tau = 1, \sigma = 1\rangle = \frac{1}{\sqrt{R_+}} \begin{pmatrix} E_+ + M_z k_z \\ Ak_+ \\ \Delta \\ 0 \end{pmatrix}. \quad (5)$$

$$|\tau = 1, \sigma = -1\rangle = \frac{1}{\sqrt{R_-}} \begin{pmatrix} E_- + M_z k_z \\ Ak_+ \\ \Delta \\ 0 \end{pmatrix}. \quad (6)$$

$$|\tau = -1, \sigma = 1\rangle = \frac{1}{\sqrt{R_-}} \begin{pmatrix} 0 \\ \Delta \\ -Ak_- \\ E_- + M_z k_z \end{pmatrix}. \quad (7)$$

$$|\tau=1,\sigma=-1\rangle=\frac{1}{\sqrt{R_+}}\begin{pmatrix}0\\ \Delta\\ -Ak_-\\ E_++M_zk_z\end{pmatrix}. \tag{8}$$

with $R_\pm=A^2k_{||}^2+\Delta^2+(E_\pm+M_zk_z)^2$, which correspond to the eigenvalues as $E_{\tau\sigma}=\tau\sigma\sqrt{M_z^2k_z^2+A^2k_{||}^2+\Delta^2}$.

Optical conductivity. The current operator along the x-direction is given as

$$J_x=\frac{e}{\hbar}\frac{\partial H}{\partial k_x}=\frac{e}{\hbar}\begin{bmatrix}0 & A & 0 & 0\\ A & 0 & 0 & 0\\ 0 & 0 & 0 & -A\\ 0 & 0 & -A & 0\end{bmatrix}=\frac{e}{\hbar}A\tau_z\otimes\sigma_x. \tag{9}$$

We label the transition matrix elements as $J^x_{\tau\sigma,\tau'\sigma'}=\langle\tau,\sigma|J_x|\tau'\sigma'\rangle$, and there are four kinds of transitions:

$$J^x_{-1,1;1,1}=\frac{eA}{\hbar}\frac{1}{\sqrt{R_+R_-}}(0\quad \Delta\quad -Ak_+\quad -E_++M_zk_z)\begin{bmatrix}0 & 1 & 0 & 0\\ 1 & 0 & 0 & 0\\ 0 & 0 & 0 & -1\\ 0 & 0 & -1 & 0\end{bmatrix}\begin{pmatrix}E_++M_zk_z\\ Ak_+\\ \Delta\\ 0\end{pmatrix}$$

$$=\frac{eA}{\hbar}\frac{2\Delta E_+}{\sqrt{R_+R_-}}. \tag{10}$$

$$J^x_{-1,1;-1,-1}=\frac{eA}{\hbar}\frac{1}{\sqrt{R_+R_-}}(0\quad \Delta\quad -Ak_+\quad -E_++M_zk_z)\begin{bmatrix}0 & 1 & 0 & 0\\ 1 & 0 & 0 & 0\\ 0 & 0 & 0 & -1\\ 0 & 0 & -1 & 0\end{bmatrix}\begin{pmatrix}0\\ \Delta\\ -Ak_-\\ E_++M_zk_z\end{pmatrix}$$

$$=\frac{eA}{\hbar}\frac{2}{\sqrt{R_+R_-}}\left(M_zk_zAk_x+iAk_yE_+\right). \tag{11}$$

$$J^x_{1,-1;1,1}=\frac{eA}{\hbar}\frac{1}{\sqrt{R_+R_-}}(-E_++M_zk_z\quad Ak_-\quad \Delta\quad 0)\begin{bmatrix}0 & 1 & 0 & 0\\ 1 & 0 & 0 & 0\\ 0 & 0 & 0 & -1\\ 0 & 0 & -1 & 0\end{bmatrix}\begin{pmatrix}E_++M_zk_z\\ Ak_+\\ \Delta\\ 0\end{pmatrix}$$

$$=\frac{eA}{\hbar}\frac{2}{\sqrt{R_+R_-}}\left(M_zk_zAk_x-iAk_yE_+\right). \tag{12}$$

$$J^x_{1,-1;-1,-1}=\frac{eA}{\hbar}\frac{1}{\sqrt{R_+R_-}}(-E_++M_zk_z\quad Ak_-\quad \Delta\quad 0)\begin{bmatrix}0 & 1 & 0 & 0\\ 1 & 0 & 0 & 0\\ 0 & 0 & 0 & -1\\ 0 & 0 & -1 & 0\end{bmatrix}\begin{pmatrix}0\\ \Delta\\ -Ak_-\\ E_++M_zk_z\end{pmatrix}$$

$$=\frac{eA}{\hbar}\frac{2\Delta E_+}{\sqrt{R_+R_-}}. \tag{13}$$

Then the sum of the transition probability is

$$P=\sum_{\tau\sigma=-1,\tau'\sigma'=1}|J^{x}_{\tau\sigma,\tau'\sigma'}|^2=\frac{e^2A^2}{\hbar^2}\frac{8}{R_+R_-}\left(M_z^2K_z^2A^2k_x^2+A^2k_y^2E_+^2+\Delta^2E_+^2\right). \quad (14)$$

By using $k_x=k_{||}\cos\phi,\ k_y=\sin\phi,\ \int_0^{2\pi}\cos^2\phi d\phi=\int_0^{2\pi}\sin^2\phi d\phi=\pi$. We have

$$\bar{P}=\int_0^{2\pi}Pd\phi=\frac{e^2A^2}{\hbar^2}\frac{8}{R_+R_-}\left[A^2k_{||}^2(M_z^2k_z^2+E_+^2)+2\Delta^2E_+^2\right]. \quad (15)$$

The optical conductance is described as

$$G_{xx}(\omega)=-\frac{1}{i\omega}\sum_{n,\mathbf{k}_{||}}P\frac{f_{n-}(\mathbf{k}_{||})-f_{n+}(\mathbf{k}_{||})}{\hbar\omega+\varepsilon_{n-}(\mathbf{k}_{||})-\varepsilon_{n+}(\mathbf{k}_{||})+i\delta}. \quad (16)$$

where $\varepsilon_{n\pm}(\mathbf{k}_{||})=\pm\sqrt{A^2k_{||}^2+M_z^2(\frac{n\pi}{L})^2+\Delta^2}$ is the energy of the *n-th* subband due to the quantization of k_z. The optical conductivity is

$$\sigma_{xx}(\omega)=-\frac{1}{i\omega}\frac{1}{4\pi^2}\sum_n\int_0^{\infty}\int_0^{2\pi}P\frac{f_{n-}(\mathbf{k}_{||})-f_{n+}(\mathbf{k}_{||})}{\hbar\omega+\varepsilon_{n-}(\mathbf{k}_{||})-\varepsilon_{n+}(\mathbf{k}_{||})+i\delta}k_{||}d\phi dk_{||}$$

$$=-\frac{1}{i\omega}\frac{1}{4\pi^2}\sum_n\int_0^{\infty}\bar{P}\frac{[f_{n-}(\mathbf{k}_{||})-f_{n+}(\mathbf{k}_{||})]}{\hbar\omega-2\varepsilon_{n+}(\mathbf{k}_{||})+i\delta}k_{||}dk_{||}. \quad (17)$$

Consider also the contribution from the time-reversal counterpart in the lower diagonal block of the Hamiltonian, the real part of the optical conductivity is

$$\mathrm{Re}\sigma_{xx}(\omega)=\frac{1}{\omega}\frac{1}{4\pi^2}\sum_n\int_0^{\infty}\bar{P}\frac{[f_{n-}(\mathbf{k}_{||})-f_{n+}(\mathbf{k}_{||})]\delta}{[\hbar\omega-2\varepsilon_{n+}(\mathbf{k}_{||})]^2+\delta^2}k_{||}dk_{||}. \quad (18)$$

The following parameters are used in the calculation[6]:

$C_0=-219\ \text{meV}\cdot\text{nm}^2,\ C_1=-300\ \text{meV}\cdot\text{nm}^2,\ C_2=-160\ \text{meV}\cdot\text{nm}^2,$

$M_0=10\ \text{meV}\cdot\text{nm}^2,\ M_1=9600\ \text{meV}\cdot\text{nm}^2,\ M_2=180\ \text{meV}\cdot\text{nm}^2,$

$\text{A}=275\ \text{meV}\cdot\text{nm},\ a_x=a_y=1.264\ \text{nm}\ ,\ a_z=2.543\ \text{nm}.$

SUPPLEMENTARY REFERENCES

1. Qu, D. X., Hor, Y. S., Xiong, J., Cava, R. J., & Ong, N. P. Quantum oscillations and Hall anomaly of surface states in the topological insulator Bi_2Te_3. *Science* **329,** 821-824 (2010).

2. Zhang, Y., Tan, Y. W., Stormer, H. L. & Kim, P. Experimental observation of the quantum Hall effect and Berry's phase in graphene. *Nature* **438,** 201-204 (2005).

3. Cao, J. *et al.* Landau level splitting in Cd_3As_2 under high magnetic fields. *Nat. Commun.* **6,** 7779 (2015).

4. Gorbar, E. V., Miransky, V. A. and Shovkovy, I. A. Engineering Weyl nodes in Dirac semimetals by a magnetic field. *Phys. Rev. B* **88,** 165105 (2013).

5. Sukhachov, P. O. Gap generation in Weyl semimetals in a model with local four-fermion interaction. Preprint at http://arxiv.org/abs/1406.6522v1 (2014).

6. Pan, H., Wu, M. M., Liu, Y., Yang, S. A. Electric control of topological phase transitions in Dirac semimetal thin films, *Scientific Reports* **5**, 14639 (2015).
